# Circulating Tumor DNA in Multiple Myeloma: Current Insights and Future Perspectives

**DOI:** 10.3390/biology15141208

**Published:** 2026-07-22

**Authors:** Aleksandra Sretenovic, Marko Mitrovic, Nikola Vukosavljevic, Natalija Kecman, Nada Kraguljac Kurtović, Marija Denčić Fekete, Jelena Bila

**Affiliations:** 1 Clinic of Hematology, University Clinical Center of Serbia, 11000 Belgrade, Serbia; markomitrovic40@gmail.com (M.M.); kecman.natalija@gmail.com (N.K.); nada_kraguljac@yahoo.com (N.K.K.); biladr.jelena@gmail.com (J.B.); 2 Medical Faculty, University of Belgrade, 11000 Belgrade, Serbia; marijadfekete@yahoo.com; 3 Department for Hemato-Oncology, University Clinical Hospital Center “Zvezdara”, 11000 Belgrade, Serbia; nvukosavljevic93@gmail.com

**Keywords:** multiple myeloma, circulating tumor DNA, liquid biopsy, clonal evolution, precision medicine

## Abstract

Circulating tumor DNA (ctDNA) has emerged as a promising minimally invasive biomarker in multiple myeloma (MM), providing dynamic insight into tumor burden, clonal evolution and molecular heterogeneity. Unlike conventional bone marrow-based diagnostics, ctDNA analysis offers a systemic approach to disease assessment and may better reflect spatially heterogeneous and extramedullary disease. Recent advances in highly sensitive molecular techniques, including digital droplet polymerase chain reaction and next-generation sequencing, have improved the feasibility of ctDNA detection and longitudinal disease monitoring in MM. Increasing evidence demonstrates substantial concordance between ctDNA and bone marrow genomic profiles, while also highlighting the ability of ctDNA to identify resistant subclones, molecular relapse and genomic evolution during therapy. ctDNA has shown potential clinical utility in molecular profiling, therapeutic monitoring and minimal residual disease assessment. ctDNA may become increasingly relevant in the era of novel immunotherapies, including chimeric antigen receptor T-cell therapy and bispecific antibodies. Despite these promising applications, several biological and technical limitations still restrict routine clinical implementation, including low ctDNA concentration in patients with minimal disease burden and lack of methodological standardization. Overall, ctDNA represents a rapidly evolving tool with significant potential to improve personalized disease monitoring and therapeutic strategies in MM.

## Simple Summary

This review summarizes current evidence on the role of circulating tumor DNA in multiple myeloma. We discuss its potential use for disease characterization, assessment of tumor burden, treatment monitoring, detection of minimal residual disease, and identification of treatment resistance. Although this approach is highly promising, several technical and biological challenges still limit its routine use in clinical practice. Further large prospective studies and standardized testing methods are needed before circulating tumor DNA can become part of everyday management of patients with multiple myeloma.

## 1. Introduction

Multiple myeloma (MM) is a biologically heterogeneous plasma cell malignancy characterized by complex genomic architecture, clonal evolution and recurrent relapses throughout the disease course [1]. Despite substantial therapeutic advances achieved with proteasome inhibitors, immunomodulatory agents, monoclonal antibodies, bispecific antibodies and chimeric antigen receptor T-cell (CAR-T) therapy, MM remains incurable for many patients. Consequently, precise disease characterization, dynamic molecular profiling and reliable monitoring strategies have become increasingly important in contemporary MM management [1].

Accurate risk stratification is therefore an essential component of MM management, as it guides treatment intensity, informs prognosis, and ensures appropriate patient selection for clinical trials evaluating novel therapeutic strategies [1]. Over the past two decades, several staging systems have been developed and refined to improve prognostic accuracy.

The International Staging System (ISS), introduced in 2005, represented the first universally accepted and globally applicable risk classification tool for MM [2]. It is based on two readily available laboratory parameters: serum β2-microglobulin, which reflects tumor burden and renal function, and serum albumin, a surrogate marker of nutritional status and systemic inflammation. Based on these variables, patients are stratified into three stages (I–III), with median overall survival ranging from more than 8 years in stage I to approximately 3 years in stage III. While the ISS offered simplicity and broad applicability, it does not incorporate cytogenetic or molecular features and therefore fails to fully capture the underlying biological heterogeneity of MM.

To overcome these limitations, the Revised International Staging System (R-ISS) was proposed in 2015 [3]. This model retained the original ISS parameters but integrated two additional factors: serum lactate dehydrogenase (LDH), a marker of proliferative disease, and the presence of high-risk cytogenetic abnormalities, specifically del (17p), t (4;14), and t (14;16), as detected by fluorescence in situ hybridization (FISH). The R-ISS significantly improved prognostic discrimination, particularly by refining risk categories within ISS stage II, which had previously included a highly heterogeneous patient population.

The Second Revision of the International Staging System (R2-ISS) was developed through international collaborative efforts and validated across several independent cohorts [4]. This refined model incorporates additional adverse cytogenetic abnormalities, most notably 1q gain or amplification and 1p deletion, both of which have been consistently associated with unfavorable clinical outcomes. The R2-ISS stratifies patients into four distinct prognostic categories (I–IV), offering improved discrimination between risk groups and more accurate survival prediction. Early validation studies demonstrated clear separation of progression-free and overall survival across all four stages, supporting the R2-ISS as a valuable tool for baseline prognostic assessment in MM.

In parallel with the development of staging systems, expert groups such as the International Myeloma Society (IMS) and the International Myeloma Working Group (IMWG) have issued consensus recommendations defining high-risk MM [5,6]. These guidelines emphasize the prognostic relevance of cytogenetic abnormalities, including del (17p), t (4;14), and t (14;16), as well as additional lesions such as 1q gain and 1p deletion, which are now recognized as adverse prognostic markers. Importantly, the consensus underscores the need to integrate clinical features, genetic risk, and depth of treatment response to identify patients who may benefit from intensified therapeutic approaches or enrollment in clinical trials exploring novel agents.

Even with these refinements, conventional risk stratification systems remain limited by their reliance on static, baseline parameters derived from single-site bone marrow sampling. Such approaches do not fully capture the spatial and temporal heterogeneity of MM, in which genetically distinct subclones may coexist across different bone marrow niches and extramedullary sites, nor do they fully capture clonal evolution during treatment [7]. These limitations highlight the need for complementary biomarkers capable of providing a more comprehensive and dynamic assessment of disease biology.

In recent years, liquid biopsy has emerged as a minimally invasive diagnostic and monitoring approach in oncology [7]. Liquid biopsy refers to the analysis of tumor-derived material released into body fluids, including circulating tumor cells (CTCs), extracellular vesicles, circulating proteins, RNA and circulating cell-free DNA (cfDNA). It is important to distinguish between CTCs, cfDNA and ctDNA. CTCs are intact, viable tumor cells circulating in peripheral blood that enable single-cell phenotypic and genomic analyses, although they are rare and technically challenging to isolate. cfDNA comprises all DNA fragments released into the bloodstream from both normal and malignant cells as a result of apoptosis, necrosis or active secretion. Only a fraction of cfDNA originates from tumor cells; this tumor-derived component, referred to as ctDNA, carries the same somatic mutations, structural variants and epigenetic alterations as the malignant clone, making it a highly specific and practical biomarker for non-invasive disease monitoring [7,8,9]. ctDNA is released into the circulation predominantly through apoptosis and necrosis of tumor cells, although active secretion has also been proposed. Its concentration is influenced by several biological factors, including tumor burden, disease localization, cellular turnover, vascularization, and treatment-induced cell death. Patients with extensive bone marrow infiltration or extramedullary disease generally exhibit higher ctDNA levels than those with minimal residual disease, which directly affects assay sensitivity and clinical interpretation.

Owing to advances in highly sensitive molecular technologies, ctDNA analysis has become increasingly feasible in clinical practice. In solid tumors, ctDNA has already demonstrated important applications in molecular profiling, treatment selection, monitoring of therapeutic response, minimal residual disease (MRD) assessment, and early detection of relapse. Similar interest has recently emerged in hematologic malignancies, including MM [7,8].

The potential role of ctDNA in MM is particularly interesting because of the biological complexity of the disease [8]. MM frequently demonstrates patchy bone marrow infiltration, focal lesions, and extramedullary dissemination, often accompanied by substantial spatial and temporal genomic heterogeneity [8]. Different anatomical disease sites may harbor genetically distinct subclones that evolve dynamically under therapeutic pressure. Therefore, single-site bone marrow biopsy, although still considered the diagnostic gold standard, may not adequately capture the full genomic complexity of the disease [8,9].

Repeated bone marrow sampling is invasive, uncomfortable for patients, and difficult to perform during long-term monitoring, especially in the era of MRD-driven therapeutic strategies [10]. Conventional laboratory parameters, including serum and urine monoclonal protein measurements, may also have limited sensitivity in oligosecretory and non-secretory disease. Consequently, there is an increasing need for minimally invasive biomarkers capable of providing dynamic, whole-body assessment of disease burden and clonal evolution [8,9,10].

Current evidence indicates substantial concordance between ctDNA and bone marrow genomic profiles in MM, while simultaneously highlighting the ability of ctDNA to identify spatial heterogeneity, resistant subclones, extramedullary disease and molecular relapse [11,12]. Elevated ctDNA levels have been associated with higher tumor burden, advanced disease stage, inferior survival outcomes and increased disease dissemination [11,13]. These findings suggest that ctDNA may represent not only a minimally invasive surrogate biomarker, but also a dynamic platform for molecular profiling and longitudinal disease monitoring.

Although currently established prognostic systems, including the International Staging System (ISS), Revised ISS (R-ISS), and second Revised ISS (R2-ISS), remain essential tools in MM risk stratification, they predominantly provide static prognostic information obtained at diagnosis [2,3,4]. In contrast, ctDNA analysis may offer real-time insight into ongoing tumor evolution and therapeutic resistance, potentially complementing existing prognostic and monitoring strategies [8].

The integration of ctDNA analysis into translational and clinical research has therefore generated considerable interest regarding its future role in routine MM management, particularly in disease characterization, therapeutic selection, response monitoring, minimal residual disease assessment and emerging immunotherapeutic approaches [8].

## 2. Aim of the Review

The aim of this review is to provide a comprehensive overview of the current clinical applications of circulating tumor DNA analysis in multiple myeloma, including its role in disease characterization, molecular profiling, treatment monitoring, minimal residual disease assessment, and emerging therapeutic strategies. In addition, this review discusses the emerging role of ctDNA in novel immunotherapeutic strategies, including cellular therapies, as well as the current limitations, technical challenges and future perspectives of ctDNA implementation in routine clinical practice.

## 3. Methodology of ctDNA Detection in Multiple Myeloma

The clinical applicability of ctDNA analysis in MM largely depends on the sensitivity, specificity and reproducibility of currently available molecular techniques. Since ctDNA usually represents only a small fraction of total circulating cell-free DNA (cfDNA), accurate detection requires highly sensitive analytical methods capable of identifying low-frequency tumor-associated genomic alterations. Recent technological advances have significantly improved the feasibility of ctDNA analysis in hematologic malignancies, enabling increasingly detailed molecular characterization and longitudinal disease monitoring [7,8,14].

### 3.1. Molecular Techniques for ctDNA Detection

Several molecular platforms are currently used for ctDNA analysis in MM, each characterized by distinct analytical sensitivities, genomic coverage and clinical applicability.

Digital droplet polymerase chain reaction (ddPCR) is among the most sensitive techniques for ctDNA detection and allows highly accurate quantification of predefined genomic alterations with very low variant allele frequencies [15]. ddPCR is particularly useful for monitoring recurrent hotspot mutations, including KRAS, NRAS, BRAF, and TP53 alterations during disease progression and treatment response assessment. However, its major limitation lies in the requirement for prior knowledge of target mutations, thereby restricting broader genomic profiling [15,16].

Next-generation sequencing (NGS)-based approaches have become increasingly important in ctDNA analysis because they enable simultaneous evaluation of multiple genomic alterations [9,14,15,16]. Targeted deep sequencing panels are commonly employed to detect recurrent MM-associated mutations and copy number abnormalities, while whole-exome sequencing (WES) and whole-genome sequencing (WGS) provide more comprehensive characterization of tumor genomic architecture and clonal evolution.

More recently, highly sensitive techniques such as Cancer Personalized Profiling by Deep Sequencing (CAPP-Seq) have demonstrated promising results in improving ctDNA detection sensitivity, particularly for minimal residual disease assessment and longitudinal monitoring [17]. CAPP-Seq can identify multiple tumor-specific mutations within an individual patient’s tumor, thereby increasing analytical sensitivity and enabling a more comprehensive characterization of the tumor genomic landscape. This approach reduces false-negative results, particularly in tumors with low ctDNA abundance. CAPP-Seq provides valuable insight into intratumoral heterogeneity, reflecting the presence of distinct tumor subclones that may differ in their biological behavior and treatment sensitivity [17].

From a clinical perspective, this is particularly relevant in multiple myeloma, where spatial heterogeneity and clonal evolution frequently contribute to disease progression and therapeutic resistance. Consequently, CAPP-Seq may improve disease monitoring and facilitate the early detection of emerging resistant clones.

Pre-analytical sample handling is an important determinant of ctDNA analysis quality. EDTA plasma is generally preferred over serum because coagulation in serum may lead to leukocyte lysis and genomic DNA contamination, thereby reducing analytical sensitivity [18].

In addition to mutation-based approaches, patient-specific immunoglobulin gene rearrangements, particularly those involving the immunoglobulin heavy-chain (IGH) locus, represent highly specific molecular targets for ctDNA analysis. Because these rearrangements are unique to each patient’s malignant plasma cell clone, they may serve as personalized biomarkers for disease monitoring and MRD assessment. This approach may further improve the sensitivity and specificity of ctDNA-based monitoring in multiple myeloma [14].

### 3.2. Concordance Between ctDNA and Bone Marrow Genomic Profiles

Published data have demonstrated substantial concordance between ctDNA and bone marrow genomic profiles in MM, supporting the role of ctDNA as a minimally invasive surrogate of tumor genomic architecture. ctDNA analysis has shown the ability to identify clinically relevant mutations, copy number alterations, and patterns of clonal evolution comparable to those detected in bone marrow plasma cells [9,14,15,16,19].

Importantly, ctDNA may additionally capture spatial genomic heterogeneity and disease dissemination beyond the bone marrow compartment [12]. Since MM frequently demonstrates patchy bone marrow infiltration and genetically heterogeneous focal lesions, single-site bone marrow biopsy may underestimate the overall tumor genomic complexity. ctDNA released from multiple disease sites into peripheral blood may therefore provide a more comprehensive representation of whole-body disease biology [12,14,16].

Discordance between bone marrow and ctDNA findings may occur, particularly in patients with low tumor burden or limited circulating tumor DNA concentrations. Consequently, ctDNA currently represents a complementary rather than fully substitutive approach to conventional bone marrow-based molecular assessment [8,14]. Key differences between conventional bone marrow biopsy and ctDNA analysis are summarized in Table 1.

During the preparation of this manuscript, the authors used ChatGPT (OpenAI, GPT-5.5) only to assist in the preparation and graphical presentation of the two tables included in this review. The authors have reviewed and edited the output and take full responsibility for the content of this publication.

### 3.3. Current Technical Limitations

Despite major technological advances, several biological and technical challenges still limit the routine clinical use of ctDNA analysis in MM. One of the major challenges is the relatively low concentration of ctDNA in certain patients, particularly during deep remission or minimal residual disease negativity [7,8,20]. Analytical sensitivity therefore remains critically important, especially for longitudinal disease monitoring.

Another important limitation is the lack of methodological standardization regarding blood collection, plasma processing, sequencing platforms, bioinformatic analysis methods and reporting criteria [7,8]. Inter-study variability currently limits direct comparison between published studies and complicates the establishment of universally accepted clinical thresholds. Beyond analytical performance, the economic implications of ctDNA testing represent an important consideration. Comprehensive ctDNA assays often involve ultra-deep sequencing and complex data analysis, resulting in higher costs compared with established diagnostic modalities. Formal cost-effectiveness analyses will therefore be necessary to assess whether ctDNA-based approaches provide sufficient clinical benefit to justify their implementation in routine care.

Although current technologies continue to improve analytical sensitivity and specificity, further prospective studies and standardized protocols remain necessary before ctDNA analysis can be fully integrated into routine clinical management of multiple myeloma [7,8].

## 4. Clinical Applications of ctDNA

### 4.1. ctDNA in Precursor States: MGUS and Smoldering MM

Monoclonal gammopathy of undetermined significance (MGUS) and smoldering multiple myeloma (SMM) represent asymptomatic precursor conditions within the spectrum of plasma cell dyscrasias, both of which carry a variable but measurable risk of progression to overt multiple myeloma [21]. While MGUS typically progresses at an average rate of approximately 1% per year, SMM is associated with a substantially higher and more heterogeneous risk of transformation, particularly within the first few years following diagnosis. Accurate identification of individuals at highest risk of progression therefore remains a major clinical challenge.

Current risk stratification models for MGUS and SMM rely primarily on clinical and laboratory parameters, including serum M-protein concentration, bone marrow plasma cell percentage, and the presence of immunoparesis [21]. Although these factors provide a general framework for estimating progression risk, they offer limited precision in predicting the timing and likelihood of progression at the individual patient level. As a result, a substantial proportion of patients categorized as intermediate risk remain clinically stable for prolonged periods, whereas others progress rapidly despite apparently favorable baseline characteristics [21].

In this context, ctDNA has emerged as a potential biomarker capable of refining risk assessment in precursor disease. Several studies have demonstrated that ctDNA is detectable in a subset of patients with MGUS and SMM, albeit at significantly lower concentrations than those observed in symptomatic MM [8]. Importantly, the presence of detectable ctDNA in these early disease stages has been associated with an increased risk of progression, suggesting that ctDNA may capture underlying biological aggressiveness not fully reflected by conventional clinical markers [8,14]. Incorporation of ctDNA into existing risk models could therefore improve the identification of patients who may benefit from closer surveillance or consideration of early interventional strategies.

Beyond quantitative assessment, ctDNA analysis allows for the detection of recurrent somatic mutations implicated in disease progression, including alterations in KRAS, NRAS, and TP53 [8,14]. The presence of such mutations in ctDNA may indicate early clonal selection and genomic instability, providing insight into the molecular events that precede clinical transformation. Importantly, longitudinal sampling of ctDNA enables dynamic tracking of clonal evolution over time, allowing detection of emerging high-risk subclones before overt progression becomes clinically evident [8,14,22]. This longitudinal approach has the potential to inform surveillance intensity and support enrollment of high-risk patients into early-phase therapeutic trials.

Despite these promising observations, several challenges currently limit the clinical application of ctDNA in MGUS and SMM. The low fraction of tumor-derived DNA in circulation, particularly in patients with minimal disease burden, presents a significant analytical challenge and increases the risk of false-negative results. In addition, variability in assay sensitivity, sequencing depth, and analytical pipelines, together with the lack of standardized methodologies, hampers reproducibility across studies [8,14,22]. Consequently, large-scale prospective studies are required to validate ctDNA as a reliable biomarker for progression risk and to define its role in guiding early intervention strategies in precursor plasma cell disorders [8,14,23].

### 4.2. ctDNA in Newly Diagnosed and Relapsed MM: Tumor Burden Assessment

One of the most extensively investigated clinical applications of ctDNA in MM is the assessment of tumor burden and disease dissemination. Recent findings have demonstrated significant correlations between ctDNA levels and established markers of disease activity, including bone marrow plasma cell infiltration, β2-microglobulin, lactate dehydrogenase (LDH) and advanced ISS and R-ISS stages. Elevated ctDNA levels have additionally been associated with inferior progression-free survival and overall survival, supporting the potential prognostic significance of ctDNA in MM [8].

Importantly, ctDNA analysis may provide a more comprehensive representation of whole-body disease burden compared with single-site bone marrow biopsy. MM is characterized by patchy bone marrow infiltration, focal lesions and substantial spatial genomic heterogeneity between different anatomical disease sites. Consequently, conventional bone marrow sampling may underestimate the overall tumor genomic complexity and fail to adequately reflect extramedullary disease involvement [12].

Recent studies have shown that increased ctDNA tumor fraction correlates with a higher number of focal lesions, metabolically active disease on 18F-FDG PET/CT, paraskeletal involvement and extramedullary dissemination [11]. Higher ctDNA levels have also been associated with inferior progression-free and overall survival, supporting its potential role as a biomarker of disease burden and aggressive disease biology. Most of the currently available evidence originates from predominantly single-center cohorts with heterogeneous methodologies and limited follow-up. Although the available data are promising, larger prospective multicenter studies are needed before ctDNA can be incorporated into routine risk stratification in multiple myeloma [8,11,14].

Integration of ctDNA levels with currently established prognostic systems may improve risk stratification and facilitate identification of biologically high-risk patients not fully recognized by conventional staging models.

### 4.3. Molecular Profiling and Treatment Selection

ctDNA analysis enables minimally invasive molecular profiling and dynamic assessment of genomic alterations relevant for therapeutic decision-making in MM [19,22,23].

One of the major advantages of ctDNA analysis is its ability to capture spatial genomic heterogeneity that may not be detected by a single-site bone marrow biopsy. Distinct disease sites may harbor genetically heterogeneous subclones with different therapeutic sensitivities and resistance mechanisms. Therefore, ctDNA may provide a more representative overview of the global tumor genomic architecture and support personalized therapeutic approaches [9,12].

As treatment strategies in hematologic oncology become more personalized, ctDNA analysis may play an increasingly important role in molecular characterization and treatment selection. Serial ctDNA monitoring may help identify emerging resistant clones and allow earlier recognition of treatment failure, potentially supporting more effective therapeutic sequencing [19,22].

### 4.4. Assessment of Treatment Response and Its Duration

Compared with traditional biochemical markers, ctDNA may provide earlier insight into molecular response dynamics and clonal evolution during therapy. Importantly, ctDNA analysis can identify molecular relapse before overt biochemical or clinical progression becomes evident. This may be particularly relevant in patients with oligosecretory or non-secretory MM, in whom conventional serologic monitoring may be insufficient [24,25].

### 4.5. Minimal Residual Disease Monitoring

Minimal residual disease (MRD) assessment has become one of the most important prognostic tools in contemporary MM management [26,27,28,29]. Current MRD assessment primarily relies on bone marrow-based next-generation flow cytometry (NGF) and next-generation sequencing (NGS), which achieve validated sensitivities of up to 10^−5^–10^−6^ and are incorporated into response-adapted therapeutic strategies and clinical trials. Because of patchy bone marrow infiltration and spatial genomic heterogeneity, bone marrow evaluation may underestimate residual disease in some patients.

ctDNA analysis represents a promising complementary strategy for non-invasive MRD monitoring. Unlike bone marrow biopsy, ctDNA may better reflect the overall tumor burden by capturing tumor-derived DNA released from multiple anatomical sites, including extramedullary lesions. In addition, serial peripheral blood sampling enables more frequent disease monitoring than repeated bone marrow aspirations.

Although several studies have demonstrated an association between ctDNA dynamics and treatment response, the reported concordance between ctDNA- and bone marrow-based MRD assessment remains variable. In particular, one study demonstrated limited concordance between these approaches, indicating that ctDNA alone cannot yet reliably replace established bone marrow MRD methodologies [30]. The relatively low ctDNA concentration observed in patients with deep remission or minimal residual disease further limits analytical sensitivity and increases the risk of false-negative results.

Consequently, ctDNA should currently be considered a complementary rather than a replacement approach for MRD assessment. Future prospective studies are needed to standardize ctDNA-based MRD methodologies and to define their optimal integration with established bone marrow MRD techniques [29].

## 5. Challenges and Future Directions

Despite the rapidly growing interest in ctDNA analysis in MM, several biological, technical and clinical challenges still limit its routine implementation [31]. One of the main limitations is the low concentration of ctDNA in peripheral blood, particularly in patients with low tumor burden, precursor plasma cell disorders or deep remission after effective therapy. In these settings, ctDNA may fall below the limit of detection, leading to false-negative results and limiting its reliability for minimal residual disease monitoring [31].

A major barrier to clinical translation is the lack of standardization across the entire ctDNA workflow. Variability in pre-analytical factors, such as blood collection tubes, processing time, plasma separation and storage conditions, can significantly affect ctDNA yield and integrity. In addition, heterogeneity in sequencing platforms, depth of coverage, variant calling strategies and reporting thresholds further compromises reproducibility and cross-study comparability. The development of standardized protocols, quality assurance measures and consensus reporting guidelines will be essential to ensure reliable interpretation and facilitate broader clinical adoption [31].

From a clinical perspective, the exact role of ctDNA alongside established diagnostic and monitoring approaches remains to be fully defined. Future studies are needed to determine how ctDNA can best complement bone marrow MRD assessment and other conventional disease monitoring methods.

Large prospective, multicenter studies are required to validate ctDNA-guided clinical decisions, define clinically meaningful thresholds, determine optimal sampling intervals and evaluate whether early therapeutic intervention based on ctDNA dynamics can improve patient outcomes. The major advantages and current limitations of ctDNA analysis in multiple myeloma are summarized in Table 2.

During the preparation of this manuscript, the authors used ChatGPT (OpenAI, GPT-5.5) only to assist in the preparation and graphical presentation of the two tables included in this review. The authors have reviewed and edited the output and take full responsibility for the content of this publication.

### Role of ctDNA in Cellular Immunotherapy

The introduction of novel immunotherapeutic strategies, including chimeric antigen receptor T-cell (CAR-T) therapy and bispecific antibodies, has significantly changed the therapeutic landscape of multiple myeloma (MM) [32,33]. These approaches have enabled deep and durable responses even in heavily pretreated patients, while simultaneously increasing the importance of highly sensitive disease monitoring methods capable of detecting residual disease and emerging therapeutic resistance.

Recent clinical evidence has demonstrated the potential value of ctDNA monitoring during CAR-T therapy. Serial ctDNA analysis may provide early information on treatment response. Persistent or increasing ctDNA levels during follow-up have been associated with poorer outcomes and may indicate disease progression before clinical relapse becomes apparent [32].

Longitudinal ctDNA profiling enabled the detection of clonal evolution, highlighting its potential role in guiding individualized post-CAR-T monitoring strategies [32].

ctDNA monitoring is expected to play an increasingly important role during treatment with bispecific antibodies. Continuous immune-mediated selective pressure imposed by these therapies may drive clonal selection and the emergence of therapy-resistant subclones. Serial ctDNA analysis has the potential to capture these dynamic genomic changes in real time, enabling earlier detection of resistance and optimization of subsequent therapeutic strategies [32,33].

Despite these promising findings, the role of ctDNA in monitoring novel cellular therapies remains investigational. Larger prospective clinical trials are required to validate the prognostic and predictive value of ctDNA dynamics during immunotherapy and to define standardized approaches for its clinical implementation.

## 6. Conclusions

Circulating tumor DNA analysis has emerged as a highly promising minimally invasive approach for disease characterization, molecular profiling and dynamic monitoring in multiple myeloma. By providing insight into clonal evolution, spatial heterogeneity and treatment-related molecular changes, ctDNA has the potential to complement conventional bone marrow-based diagnostics and improve longitudinal disease assessment.

Recent technological advances have substantially improved the sensitivity and feasibility of ctDNA detection, enabling increasingly detailed evaluation of treatment response, molecular relapse and minimal residual disease. In particular, ctDNA may offer important advantages in patients with extramedullary, oligosecretory or non-secretory disease, where standard monitoring approaches often have important limitations.

Beyond disease monitoring, ctDNA may also contribute to more refined risk stratification and individualized therapeutic decision-making by enabling serial assessment of genomic evolution throughout the disease course. As treatment strategies continue to evolve toward precision medicine, real-time molecular profiling may facilitate earlier identification of resistant subclones, optimization of treatment sequencing and more personalized therapeutic adaptation.

Despite encouraging results, several challenges still prevent routine clinical implementation. Limited ctDNA concentration in patients with low disease burden, methodological heterogeneity and the absence of standardized analytical workflows remain major obstacles. Furthermore, most currently available evidence originates from relatively small and heterogeneous patient cohorts, highlighting the need for large prospective multicenter studies.

Rather than replacing bone marrow biopsy, ctDNA is likely to become an important complementary component of integrated disease assessment. Future monitoring strategies will probably combine ctDNA analysis with bone marrow MRD evaluation, advanced imaging techniques, circulating tumor cells and other emerging biomarkers to provide a more comprehensive and dynamic understanding of disease biology.

Overall, ctDNA represents one of the most promising advances in liquid biopsy for multiple myeloma. As molecular technologies continue to mature and analytical approaches become standardized, ctDNA has the potential to become an integral part of precision medicine, enabling more individualized treatment decisions and improving disease monitoring throughout the entire course of multiple myeloma.

## Figures and Tables

**Table 1:** Comparison Between Bone Marrow Biopsy and ctDNA Analysis in MM.

Feature	Bone Marrow Biopsy	ctDNA Analysis
Sample source	Bone marrow aspirate/biopsy	Peripheral blood
Invasiveness	Invasive procedure	Minimally invasive
Disease representation	Single-site assessment	Systemic disease assessment
Spatial heterogeneity assessment	May miss heterogeneous lesions	Better reflects spatial heterogeneity
Extramedullary disease detection	Limited sensitivity	Potentially more informative
Repeatability	Patient discomfort	Easily repeatable
Molecular profiling	Standard diagnostic approach	Complementary molecular assessment
MRD monitoring	Established standard methods available	Still investigational/complementary
Main limitations	Sampling bias, invasiveness	Low ctDNA levels, lack of standardization

**Table 2:** Advantages and Limitations of ctDNA Analysis in Multiple Myeloma.

Advantages	Limitations
Minimally invasive approach based on peripheral blood sampling	Low ctDNA levels in patients with low tumor burden or deep remission
Enables serial and longitudinal disease monitoring	Limited sensitivity for MRD detection in some patients
Reflects spatial heterogeneity and extramedullary disease	Lack of methodological standardization
Provides dynamic insight into clonal evolution	Variability in sequencing platforms and analytical methods
Allows early detection of resistant subclones and molecular relapse	Technical complexity and cost
Potentially complements bone marrow-based diagnostics	Requires highly sensitive molecular techniques

## Data Availability

No new data were created or analyzed in this study. Data sharing is not applicable to this article.

## Abbreviations

The following abbreviations are used in this manuscript:

CAPP-Seq	Cancer Personalized Profiling by Deep Sequencing
CAR-T	Chimeric antigen receptor T-cell
cfDNA	Cell-free DNA
CTC	Circulating tumor cell
ctDNA	Circulating tumor DNA
ddPCR	Digital droplet polymerase chain reaction
FISH	Fluorescence in situ hybridization
IGH	Immunoglobulin heavy chain
IMWG	International Myeloma Working Group
IMS	International Myeloma Society
ISS	International Staging System
LDH	Lactate dehydrogenase
MGUS	Monoclonal gammopathy of undetermined significance
MM	Multiple myeloma
MRD	Minimal residual disease
NGF	Next-generation flow cytometry
NGS	Next-generation sequencing
PCR	Polymerase chain reaction
R-ISS	Revised International Staging System
R2-ISS	Second Revision of the International Staging System
RNA	Ribonucleic acid
SMM	Smoldering multiple myeloma
WES	Whole-exome sequencing
WGS	Whole-genome sequencing
